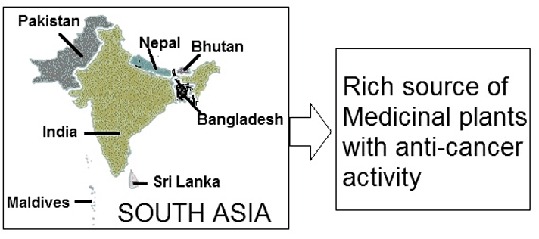# Anti-cancer potential of South Asian plants

**DOI:** 10.1007/s13659-013-0027-6

**Published:** 2013-05-15

**Authors:** Mohammad Mijanur Rahman, Md. Asaduzzaman Khan

**Affiliations:** 127 Department of Biochemistry and Molecular Biology, Jahangirnagar University, Savar, Dhaka, 1342 Bangladesh; 227 Department of Biochemistry, School of Life Sciences, Central South University, Changsha, 410013 China

**Keywords:** natural products, South Asia, medicinal plants, anti-cancer activities, phyto-chemicals

## Abstract

Phyto-chemicals are increasingly being used in the treatment of cancer because of their availability, potential anti-cancer activity with less adverse effects when compared with chemotherapy. The variation of climate and geography in South Asian countries provides a nursing environment for the growth of versatile plant species, that are repeatedly drawing attention of the scientific community. In this review, we have focused on the anti-cancer potential of thirty plants, which are commonly found in Bangladesh, India, Nepal, Pakistan and Sri Lanka, with their mechanisms of action. In particular, we have discussed the bio-active components that display anti-cancer activity, which have been identified in these plants. This review may help researchers to profile plants with known anti-cancer effect of this region and further investigations of anti-cancer agents in medicinal plants from South Asia.

## 1 Phyto-chemicals, as Anti-cancer Agents

In the last century, significant development in bio-medical science has conquered many diseases, however cancer remains ambiguous especially from a therapeutic perspective. Cancer is still a growing health problem world-wide, and it is the second most common cause of death from disease after myocardial infarction. Tumor or neoplasm is usually defined as a growth of an abnormal mass of tissue due to uncontrolled cellular growth, while cancer is the term of all malignant tumors[1]. Non-lethal genetic damage by multi-step carcinogenesis allows for the neoplastic transformations, such as self-sufficiency in growth signal, insensitivity to growth-inhibitory signal, evasion of apoptosis, limitless replication, sustained angiogenesis and the ability to invade and metastasize[2]. However, cancer to some degree is a preventable disease, as cancer risk can be reduced by avoidance of cancer-causing biological, chemical, and physical agents, in addition to the habitual consumption of cancer protective foods[3]. Current research emphasis focuses on synthetic chemotherapeutic drugs but unfortunately conventional chemotherapy with synthetic drugs evoke severe side effects.

There are thousands of scientific studies that have focused on the pharmacological activity of bio-active components from plants, increasing interest from scientific community as cancers suppressant. Biological targets of phyto-chemicals in mammalian cells were found to be involved in inflammatory processes and oncogenic transformation, such as the alteration of cell cycle control, apoptosis evasion, angiogenesis and metastases[4]. Additionally, epidemiological studies suggest that the daily intake of certain phyto-chemicals can reduce the incidence of several types of cancers[5]. Thus, chemoprevention by dietary phyto-chemicals alone immerges as one of the most promising approaches for reduced risk of cancer development. On the other hand, phyto-chemicals act in synergy with chemotherapeutic drugs to overcome cancer cell drug resistance, and further application of specific phyto-chemicals may also allow the use of lower concentrations of drugs in cancer treatment with an increased efficacy[6]. Interestingly, according to WHO, approximately 80% of the world's inhabitants rely on traditional plant derived medicine for their primary health care[7]. Investigation of the underlying pharmacokinetic mechanisms through which phyto-chemicals evoke their anti-cancer effect introduces a panel of molecular targets. This panel includes apoptotic proteins (i.e. caspases, bax etc), protein kinases (i.e., PKA, PKC, MAPK, TYK2 etc), anti-apoptotic proteins (i.e., bcl2, TRAF1, Survivin etc), Growth factors (i.e., TNF, EGF, FGF, PDGF etc), transcription factors (i.e., Ap1, NF-κB, Nrf2, p53 etc), cell adhesion molecules (i.e., ICAM-1, VCAM etc) and cell cycle proteins (i.e., Cyclin D, CDK1, CDK2, p27, p21 etc). Moreover, phyto-chemicals interfere with multiple cell-signaling pathways[8]. A schematic diagram of the common anti-cancer mechanisms of phyto-chemicals has been depicted in Figure 1.

In South Asia, the extraordinary variation in climate and geography provides a vast spectrum of environmental conditions for the growth of versatile plant species, that are repeatedly drawing attention from the scientific community. In

*To whom correspondence should be addressed. E-mail: asadkhanbmj@yahoo.com

**Figure 1. Possible modes of action executed by phyto-chemicals against cancer.** Multi-step carcinogenesis through cancer-causing agents propels normal cells to divide uncontrollably by apoptosis evasion. The ultimate result is the neoplastic transformation of normal cells. Additional tumor progression through clonal proliferation, immune evasion and angiogenesis leads non-metastatic tumor that usually metastasizes to a secondary site after the loss of adhesiveness and diversification. Preventive and anti-mutagenic phyto-chemicals halt or retard the progression of normal and precancerous cells respectively into malignant, or reverse the promotion stage of multi-step carcinogenesis. Anti-proliferative phyto-chemicals inhibit cell growth and proliferation while apoptosis inducer phyto-chemicals induce apoptosis of neoplastic cells. Another pronounced impact of phyto-chemicals is the prevention of metastasis and angiogenesis[2,3,8].

this review, we have focused on the anti-cancer potential of certain herbal plant species which are commonly grown in South Asian countries such as, Bangladesh, India, Nepal, Pakistan and Sri Lanka. We have searched databases such as Pubmed, Google Scholar, Hinari and AGORA for the basic and clinical research publications relating to the anti-cancer effects of these plants. In this review, we have included only those plants, which are commonly found in a broad spectrum range of South Asia, and excluded plant species, which have narrow spectrum of habitat.

## 2 South Asian Plants with Anti-cancer Activities

Different species of plants from South Asian countries have been reported to have anti-cancer potential (Table 1). Some of these plants are endemic world-wide, while some are strictly native.

**Table 1. Summary of the anti-cancer activities of some selective South Asian plants**

| name of the plants | bio-active compounds | experimental models | mechanisms of action | ref. |
|---|---|---|---|---|
| *Abrus precatorius* L. | protein fraction of seed extract, abrin-a, and abrin-b | Yoshida sarcoma, Fibrosarcoma, DLA, A549, Sarcoma 180 and EAC | cytotoxicity, immune modulation (agglutination by antibody), growth inhibition | (9–11) |
| *Aegiceras corniculatum* L. | aqueous extract, embilins (5-*O*-ethylembelin and 5-*O*-methylembelin) | AGS, HT-29, MDA-MB-435S, HL-60, Bel-7402, U937 and HeLa | cytotoxicity, G0/G1 cell cycle arrest, microtubule network dissemination | (12–15) |
| *Alangium salviifolium* Wang. | non-polar extract, 27-*O*-*trans*-caffeoylcylicodiscic acid, myriceric acid-B and 3-*O*-demethyl-2-*O*-methylalangiside | EAC, MOLT-3, HepG2 | cytotoxicity, growth inhibition | (17,18,21) |
| *Amoora rohituka* Roxb. | organic solvent extracts, amooranin, rohitukine | DLA, EAC, MCF-7, MDA-468, MCF-7/TH,CEM/VLB, SW620/Ad-300, non-Hodgkin's lymphoma, renal, prostrate, colon and gastric cancer cell lines | cytotoxicity, cell cycle arrest G2/M phase, caspase-activated apoptosis, growth inhibition through cyclin-dependent kinases (CDK2 and CDK1) | (22,23,25–27) |
| *Blumea lacera* L. | methanol extract, thymoquinol dimethyl ether, *β*-caryophyllene, *α*-humulene and *E*-*β*-farnesene | AGS, HT-29, MDA-MB-435S, K562, L1210, P3HR1 and U937 | cytotoxicity, reactive oxygen species induced cytotoxicity | (12,28,30–31) |
| *Bruguiera gymnorrhizais* L. | polar extracts, *ent*-kaurane diterpenoids and pimaren diterpenoids | AGS, HT-29, MDA-MB-435S, L-929 and K562 | cytotoxicity | (12,32–34) |
| *Calotropis procera* | organic extracts and latex, heat-stable latex proteins and cardiotonic steroid UNBS1450 | HL-60, HCT-8, B-16/F10, Sarcoma 180 and Hepatocellular carcinoma | cytotoxicity by apoptosis, disorganization of cytoskeleton, induction of autophagy-related cell death, repression of NF-κB, c-Myc down-regulation | (35–38) |
| *Cuscuta reflexa* Roxb. | aqueous and organic (chloroform and ethanol) extract, Cuscutin, amarbelin, *β*-sterol, stigmasterol, kaempferol, dulcitol, myricetin, quercetin, coumarin and oleanolic acid | EAC, Hep3B | cytotoxicity, p53 and Bax up-regulation, Bcl-2 and survivin down regulation | (40–42) |

| | | | | |
|---|---|---|---|---|
| *Dendrophthoe falcata* (L.F.) Ettingsh | hydroalcoholic and ethanol extract, quercetrin, catechin, gallic acid, chebulinic acid, oleonolic acid, $\beta$-amyrin-0-acetate, leucocynidin, $\beta$-sitosterol and stigmasterol | MCF-7, EAC and DMBA-induced breast carcinoma | cytotoxicity | (43,44,46) |
| *Dioscorea bulbifera* L. | petroleum ether fraction, kaempferol-3,5-dimethyl ether, caryatin, (L)-catechin, myricetin, quercetin-3-*O* galactopyranoside, myricetin-3-*O*-galactopyranoside, myricetin-3-*O*-glucopyranoside and diosbulbin B | HepA, cervical carcinoma U14, S180 and H22 | apoptosis induction, immune modulation | (48–51) |
| *Embelia ribes* Burm. F. | embelin, quercitol, flavonoids, tannins, saponins and alkaloid | Diethyl-nitrosa-mine/phenobarbital induced Hepatocarcinoma | antimutagenic effect, apoptosis induction, cell cycle arrest at in sub G1 phase, down-regulation of Bcl-2, Bcl-xL, survivin, IAP-1, IAP-2, cyclin D1, Caspase-3 activation. | (52–56) |
| *Ficus benghalensis* L. | methanol extract, lupeol, psoralen and $\beta$-sisterol | HepG2 and MCF-7 | cytotoxicity | (58–62) |
| *Ficus religiosa* L. | polar extract, quercetin and qyricetin, stigmasterol and $\beta$-sitosterol | HT29, MDA-MB-435S, MCF-7 | cytotoxicity | (12,63,64) |
| *Hibiscus tiliaceus* Linn. | aqueous extract, stigmasterol $\beta$-sitosterol, quercetin, kaempferol | DAL, Gastric cancer and Colon cancer | cytotoxicity | (12,65–67) |
| *Jatropha gossypiifolia* L. | lignans, falodone and jatrophone | A-549 ,P338 lym-phocytic leukemia | anti-proliferative effect, cytotoxicity | (68–71) |
| *Kaempferia parviflora* Wall. | ethanol extract, 7,4-trimethoxyflavon, 3,5,7,4′-tetramethoxyflavone, 5,7,4′-trimethoxyflavone and 5-hydroxy-3,7,3′,4′-tetramethoxyflavone | HL-60, HuCCA-1, RMCCA-1 and HCT-15 | induction of apoptosis, caspase activation | (72–74) |
| *Lantana camara* L. | camaraside and lantadene-A, | HL-60 | cell proliferation inhibition, caspase-3 dependant apoptosis, Bcl-2 down regulation and Bax up-regulation | (76,77) |
| *Leea indica* (Burm.f.) Merr. | alcoholic (methanol and ethanol) extract, mollic acid arabinoside and mollic acid xyloside | EAC, Ca Ski, MCF-7, MDA-MB-435, KB, HEP G2 and WRL 68 | growth inhibition, proliferative cell nuclear antigen expression decrease, S- and G2/M- phases cell cycle arrest | (78–80) |

| | | | | |
|---|---|---|---|---|
| *Mollugo pentaphylla* Linn. | apigenin and mollupentin, mmollugogenol A, mollugogenol B, mollugogenol D, oleanolic acid and *β*-sitosterol | DMBA-induced cancer, Sarcoma-180 | cytotoxicity | (81–83) |
| *Murraya koenigii* Linn. | nnon-polar extract, mahanine, mahanimbicine and mahanimbine, terpenoids | DAL, MCF-7, HeLa, P388, MOLT-3 and HL-60 | apoptosis induction by extrinsic and/ or intrinsic pathway | (84–87) |
| *Nelumbo nucifera* Willd. | liensinine, neferine, pronuciferine, isoliensinine, negferine, asimilobine, nuciferine, remrefidine, isoliensinine, flavonoids, flavonol, myricetin, quercetin, leucocyanidin, kaempferol, astragalin | EAC, HBL-100, Calu-6 human pulmonary carcinoma and SMU-601 and Human osteosarcoma cells | inhibition of growth, cytotoxicity, p21-dependent cell cycle arrest at G1 phase (anti-proliferative effect) | (88–91) |
| *Nyctanthes arbor-tristis* L. | alcoholic (ethanol and methanol) extract, glycosides including iridoid glycosides, phenylpropanoid glycosides, carotenoid glucosides, phenylpropanoid glycoside cardiac glycosides, polysaccharides, *β*-sitosterol, *β*-amyrin, hentri-acontane benzoic acid, nyctanthic acid, friedelin, lupeol, oleanolic acid, 6*β*-hydroxylonganin alkaloids, phlobatanins, terpenoids | DAL, MDA-MB 231 breast cancer cell | cytotoxicity | (92–95) |
| *Oxalis corniculata* Linn. | ethanol extract | EAC | growth inhibition | (96) |
| *Physalis minima* L. | chloroform extracts, withanolides | T-47D, Caov-3, NCI-H23, HCT-116 and NCI-H460 | growth inhibition by apoptotic pathways, autophagic cell death | (97–100) |
| *Pterocarpus santalinus* L.f. | methanol extract, melanoxoin, *S*-3-hydroxy-4,4-dimethoxydalbergione, pterolinus Hb, pterolinus-B, pterolinus-D | HeLa, Ca9-22, Hep3B, MDA-MB-231, A549 and MCF7 | apoptosis induction, chromatin condensation, DNA fragmentation, Sub-G1 phase cell accumulation | (101–103) |
| *Salix tetrasperma* Roxb. | organic extract, tannins, *β*-amyrin, lupeol, chalcinasterol, *β*-sitosterol, stigmasterol | EAC | cytotoxicity | (104,105) |
| *Solanum nigrum* Linn. | crude polysaccharides, polyphenols, gentisic acid, luteolin, apigenin, kaempferol, *m*-coumaric acid, anthocyanidin, Lunasin | HepG2, AU565,U14, mammalian cells and skin cancer | induction of apoptosis and/or autophagocytosis, inhibition H3 and H4 acetylation inhibition, Rb protein phosphorylation | (106–109) |

| | | | | |
|---|---|---|---|---|
| *Tinospora crispa* | crude polar extract, Tinoscorside A, N-formylanonaine, N-formyldehydroanonaine, N-formylnomuciferine, N-demethyl-N-formyldehydronornuciferine, Magnoflorine, Paprazine, N-trans-feruloyltyramine, N-formylasimilobine 2-*O*-*β*-D-glucopyranoside | MCF-7, HeLa, HepG2, Caov-3, MCF-7, MDA-MB-231, HeLa and 3T3 | inhibition of prolifration | (110–112) |
| *Tragia involucrate* L. | hexane and ethyl acetate extracts, flavonoids, terpenoids | EAC | growth inhibition | (113,114) |
| *Vitex negundo* L. | organic (chloroform, acetone and ethanol) extract, vitexicarpin, 6-hydroxy-4-(4-hydroxy-3-methoxyphenyl)-3-hydroxymethyl-7-methoxy-3), 4-dihydro-2-naphthaldehyde | L-929, DAL, breast, prostate and ovarian cancer cells | cytotoxicity, caspase-activated apoptosis induction | (115–118) |

**2.1** ***Abrus precatorius*** **L.:** *Abrus precatorius* L. is a deciduous dextrose climber with slender flexible branches. It is commonly known as 'Indian liquorice' and belongs to the Fabaceae family. *A. precatorius* is known in local languages as 'Kunch' in Bangladesh, 'Guncha' in India, 'Olida' in Sri Lanka, 'Ratigedi' in Nepal and 'Ghunchchi Surkh' in Pakistan. The early finding of anti-cancer activity of *A. precatorius* comes from the study by Reddy and Sirsi[9]. They identified a protein, isolated from the seeds of *A. precatorius*, which exhibited anti-tumor activity against Yoshida sarcoma in rats and fibrosarcoma in mice. They also observed an association of the anti-tumor activity with a cytotoxic effect on the tumor cells[9]. In a short term toxicity study using Dalton's lymphoma ascities (DLA) cells, *A. precatorius* seed extracts showed anti-tumor activity. The crude extract of *A. precatorius* seeds and its fractions also displayed cytotoxicity against small cell lung carcinoma (A549 cell line)[10]. Two varieties of this toxic protein (Abrin-a, and Abrin-b) were purified from the seeds of *A. precatorius*, which were similar but not identical[11]. Interestingly, Abrin-a, and Abrin-b agglutinated Sarcoma 180 cells and Ehrlich ascites tumor cells *in vitro*. It was postulated that Abrin-a, and Abrin-b might have ameliorate immune responses towards target cells through agglutination associated anti-body response. Moreover, both of these two proteins could have inhibited the growth of Ehrlich ascites tumor cells at a sub-lethal dose when tumor cells were injected simultaneously with these proteins *in vivo*[11].

**2.2** ***Aegiceras corniculatum*** **L.:** *Aegiceras corniculatum* L. is a species of shrub or tree mangrove in the Myrsinaceae family. Its distribution is in the coastal and estuarine areas of a region ranging from India, Sri Lanka, Bangladesh through South East Asia to southern China, New Guinea and Australia. 'Black Mangrove' is the common name of *A. corniculatum*. It is locally known as 'Khalsi' in Bahgladesh, 'Heen kadol' in Sri Lanka and 'Halsi' in India. Aqueous extract of *A. corniculatum* showed selective cytotoxicity against human gastric adenocarcinoma cell line AGS, human colorectal adenocarcinoma cell line HT-29, and human breast ductal carcinoma cell line MDA-MB-435S AGS, HT-29 and MDA-MB-435S[12]. Investigation of the chemical constituent showed the presence of several anti-cancer phyto-chemicals in the stems and twigs of the mangrove plant, *A .corniculatum*, of which embilins are known to have chemopreventive effects[13]. Both, 5-*O*-ethylembelin and 5-*O*-methylembelin are promising anti-mitotic and anti-cancer molecules, which target microtubular proteins. These molecules showed *in vitro* cytotoxicity against the HL-60, Bel-7402, U937, and HeLa human cancer cell lines[14]. Mechanistically, 5-*O*-ethylembelin (**1**) and 5-*O*-methylembelin (**2**) can induce G0/G1 cell cycle arrest while completely disassemble the microtubule network in HL-60 cells[15].

**2.3** ***Alangium salviifolium*** **Wang.:** *Alangium salviifolium* Wang. is a deciduous, rambling shrub or a tree belonging to the family Alangiaceae. 'Sage-leaved alangium' is the common name of *A. salviifolium*, and it is locally known as Ankor Kanta (Bangladesh), Angol (India) and Ruk anguna (Sri Lanka). Reports propose its use as an anti-diabetic, anti-epileptic, analgesic and anti-inflammatory, in particular the activities of the polar (chloroform-, ethanol-, and aqueous-) extracts of *A. salvifolium* seeds[16]. Chloroform extract of *A. salvifolium* flower displays anti-tumor effect against Ehrlich ascites carcinoma (EAC) in mice[17]. Intra-peritonial administration of the non-polar extracts of *A. salvifolium* flower at a dose of 10 mg/Kg, mimicked the anti-cancer activity of bleomycine in EAC bearing mice[18]. Biologically active ingredients of *A. salvifolium* include flavinoids, glycosides, alkaloids and saponins[19,20]. Nine protoberberine alkaloids have been isolated from *A. salviifolium*, among which, 27-*O*-*trans*-caffeoylcylicodiscic acid (**3**) and myriceric acid B (**4**) exhibited cytotoxic activity towards the MOLT-3 cell line, while 3-*O*-demethyl-2-*O*-methylalangiside (**5**) selectively inhibited the growth of the HepG2 cancer cell line[21].

**2.4 *Amoora rohituka* Roxb.:** *Amoora rohituka* Roxb., is a species of the Meliaceae family, and commonly known as 'Rohituka'. It is locally known as 'Royna' in Bangladesh, and 'Harin-hara' in India. Both *in vivo* and *in vitro* investigation flourished the anti-cancer effect of *A. rohituka*. Organic solvent extracts of *A. rohituka* bark have been reported to have significant anti-tumor activity against DLA and EAC in mice, and further its selective cytotoxic activity against breast cancer cell line MCF-7 and DLA cells[22,23]. Amooranin, a triterpenic acid extracted from the bark of *A. rohituka* trees, has significant anti-cancer potential against N-nitrosomethyl urea-induced mammary adenocarcinoma in rats and SW620 human colon carcinoma xenograft in mice[24]. The cytotoxicity of Amooranin against a panel of cancerous cell line including human mammary carcinoma MCF-7, breast carcinoma MDA-468, multi-drug resistant breast carcinoma MCF-7/TH, multi-drug-resistant leukemia (CEM/VLB), colon carcinoma (SW620/Ad-300) provides an insight to the possible mode action of *A. rohituka*. Cell cycle arrest at G2/M phase and induction of apoptosis via caspase activation pathway are likely to contribute to the cytotoxicity of amooranin[25,26]. Furthermore, flavopiridol (**6**) is a novel semi-synthetic flavone analogue of rohitukine (**7**), chromone alkaloid isolated from an *A. rohituka*, that possess significant anti-cancer activity against non-Hodgkin's lymphoma, renal, prostrate, colon, and gastric cancers. It inhibits the tumor growth via cyclin-dependent kinases (CDK2 and CDK1) inhibition and inducing apoptosis in cancerous cells[27]. Therefore, rohitukine might have similar or partial mechanism to exert its anti-cancer effects of *A. rohituka*.

**2.5 *Blumea lacera* L.:** *Blumea lacera* L., is an annual flowering herb, and belongs to Asteraceae family. 'Malay Blumea' is the common name of *B. lacera*, and is locally known as Kukursanga (Bangladesh), Kudaraundha (India) and Kukukuruduru (Sri Lanka). Methanolic extract of *B. lacera* leaves have showed non-selective cytotoxic activity against human gastric adenocarcinoma cell line AGS, human colorectal adenocarcinoma cell line HT-29, and human breast ductal carcinoma cell line MDA-MB-435S[12]. A broad spectrum anti-leukemic activity of *B. lacera* has also been documented against K562, L1210, P3HR1 and U937 leukemia cells[28]. Several phyto-constituents have been separated from the leaves of *B. lacera*, including 5-hydroxy-3,6,7,3',4'-pentamethoxy flavone and 5,3',4'-trihydroxy flavone[29]. However, the main anti-cancer effect may be within the essential oil composition of leaves. Essential oil composition of leaves include thymoquinol dimethyl ether, *β*-caryophyllene, *α*-humulene and *E*-*β*-farnesene[30]. The *α*-humulene is an anti-cancer compound which acts by reactive oxypen species induced cytotoxicity, while *β*-caryophyllene potentiates anti-cancer activity of *α*-humulene[31].

**2.6 *Bruguiera gymnorrhiza* (L.) Lam.:** *Bruguiera gymnorrhizais* (L.) Lam. is commonly known as 'Large-leafed orange mangrove' and belongs to the Rhizophoraceae family. It is known as Kankra (Bangladesh), Malkadol (Sri Lanka), and Kandal (India) as well as some local dialect names of *B. gymnorrhizais*. Methanol extract of the bark of *B. gymnorrhizais* has been reported to have anti-tumor activity in tumor bearing mice[32]. Different organic extracts of *B. gymnorrhiza* leaves have low to moderate cytotoxic activity but are selective against human cancer cell lines AGS, HT-29 and MDA-MB-435S[12]. Among several types of phyto-chemicals, the role of terpenoids were found to be prominent as anti-cancer agents[33]. Ent-kaurane diterpenoids and pimaren diterpenoids**,** isolated from stem of *B. gymnorrhiza* showed moderate cytotoxic activities against L-929 and K562 cancer cell lines[34].

**2.7 *Calotropis procera*:** *Calotropis procera*, an erect shrub, commonly known as 'Apple of Sodom'. It is a species of flowering plant in the Asclepiadaceae family, and locally known as Akanda (Bangladesh), Akado (India), Wara (Sri Lanka). The organic extracts of stems *C. procera* have been reported to have cytotoxicity against human leukemia cell line HL-60, human colon cancer cell line HCT-8 and murine melanoma cell line B-16/F10. Stem organic extracts also possess anti-tumor activity against mice bearing Sarcoma 180 tumors[35]. The chemopreventive effects of *C. procera* latex is reported through the inhibition of different mediators of inflammation with selective cytotoxicity independent of intrinsic pathway of apoptosis against hepatocellular carcinoma in the X15-myc transgenic mouse model[36]. To reveal the probable phyto-chemicals responsible for anti-cancer effect, focus has been directed towards heat-stable latex proteins from *C. procera* due to its inhibitory activities on sarcoma 180 growth[37]. On the other hand, studies have also emphasized non-protein phyto-constituent such as the cardiotonic steroid UNBS1450 (**8**) from *C. procera* that additionally exert anti-cancer activity by cytoskeleton disorganization, autophagy-related cell death induction, NF-κB activation repression as well as c-Myc down-regulation in cancer cells[38].

**2.8 *Cuscuta reflexa* Roxb.:** *Cuscuta reflexa* Roxb., is a golden yellow, leafless, parasitic herb of Convolvulaceae family, which is locally known as Swarnalata (Bangladesh), Amarvela (India), Aftimiun (Pakistan), Aga mula nethi wel (Sri Lanka) and Amar bel. A wide range of pharmacological activity of *C. reflexa* exist such as its anti-spasmodic, anti-steroidogenic, anti-hypertensive, muscle-relaxant, cardiotonic, anti-viral, anti-convulsant, hypoglycemic, anti-fertility, hypotensive, anti-inflammatory and anti-epileptic, as well as anti-cancer activities have been documented[39]. In a study, chloroform and ethanol extracts of *C. reflexa* exhibited significant anti-tumor activity in EAC-bearing mice that was comparable to the effect of 5-fluorouracil[40]. Another study reported that water extract of *C. reflexa* induced apoptosis in Hep3B cells through the up-regulation of pro-apoptotic p53, Bax and down regulation of anti-apoptotic Bcl-2 and survivin[41]. The phyto-chemical constituents isolated from *C. reflexa* include cuscutin, amarbelin, *β*-sterol, stigmasterol, kaempferol, dulcitol, myricetin, quercetin, coumarin and oleanolic acid, among which, stigmasterol, kaempferol, myricetin, and quercetin are known to have anti-cancer activity[42].

**2.9 *Dendrophthoe falcata* (L.F.) Ettingsh:** *Dendrophthoe falcata* is a perennial, climbing, woody parasitic plant of the family Loranthaceae. The common name of *D. falcata* is 'Honey Suckled Mistletoe' but is locally known as Delum pilia (Sri Lanka) and Bajrangi (Bangladesh) etc. *D. falcate*

imparts anti-cancer activity as evidenced in cancer cell lines and animal models. Ethanolic and aqueous extracts from the stem of *D. falcata* possess high cytotoxic activities against human breast cancer cells (MCF-7)[43]. Ethanol extract of *D. falcate* displayed potent anti-cancer activity against EAC-bearing mice. This anti-cancer activity was found similar to that of cisplatin[44]. Hydroalcoholic extract of *D. falcata* showed a protective role against DMBA (7,12-dimethylbenz(a)anthracite)-induced breast carcinogenesis with a concomitant amelioration of antioxidant status. Thus, antioxidant activity of *D. falcata* extract might partially contribute to the observed anti-cancer activity, in addition to the cytotoxic effect[45]. The phyto-chemical constituents of different part of *D. falcate* include flavonoids (quercetrin, catechin) tannins (gallic acid, chebulinic acid) oleonolic acid, $\beta$-amyrin-0-acetate, leucocynidin and sterols ($\beta$-sitosterol and stigmasterol) some of which have been documented as anti-cancer components[46].

**2.10 *Dioscorea bulbifera* L.:** *Dioscorea bulbifera* L. belongs to the Dioscoreaceae family and commonly known as 'Air potato'. Its local names are Lota-bori (Bangladesh), Karukanda (India), Zaminek (Pakistan), and Udala (Sri Lanka). *D. bulbifera* rhizome is used for treatment of leprosy and tumors in Bangladesh within an ethno-botanic frame[47]. Investigations showed anti-cancer activity of *D. bulbifera* in several cancers. Various impacts at the molecular level were observed regarding different *D. bulbifera* organic extracts treatment. Petroleum ether fraction of *D. bulbifera* showed potential effect against HepA with microstructure abnormality of HepA cells surface[48]. Cui[49] reported the modulation of immune response and induction of apoptosis by crude poly-saccharides of *D. bulbifera* against cervical carcinoma in U14-bearing mice. Immune system modulation might be related to anti-tumor effects of *D. bulbifera*, as reported in S180 and H22 tumor cells bearing mice[50]. Several phyto-chemicals isolated from *D. bulbifera* are considered as active principle for anti-cancer activity, including kaempferol-3, 5-dimethyl ether, caryatin, (L)-catechin, myricetin, quercetin-3-*O*-galactopyranoside, myricetin-3-*O*-galactopyranoside, myricetin-3-*O*-glucopyranoside and diosbulbin B[51].

**2.11 *Embelia ribes* Burm. F.:** *Embelia ribes* Burm. F. is commonly known as 'False black pepper', and belongs to the Myrsinaceae family. It is locally known as Biranga (Bangladesh), Bidang (India), Walangasa (Sri Lanka), and Baobadang (Pakistan). A methanol extract of *E. ribes* fruits showed protective effect against multi-mutagenicity and genotoxicity, induced by cyclophosphamide in mice, as multi-mutagenicty is considered as a molecular basis of cancer[52]. *E. ribes* contains embelin, quercitol (polyphenols), flavonoids, tannins, saponins and alkaloid[53,54]. Embelin (EB), a phenolic compound, is protective against diethyl-nitrosamine/phenobarbital induced hepatocarcinogenesis in rat[55]. Embelin also has the capacity to protect against acetic acid induced ulcerative colitis in rats[56]. This compound exerts its anti-cancer activity by cytotoxicity toward cancer cells through the induction of apoptosis, characterized by the accumulation of cells in the sub G1 phase, positive annexin-V binding, down-regulation of anti-apoptotic (Bcl-2, Bcl-xL, survivin, IAP-1, and IAP-2) and proliferative (cyclin D1) proteins, activation of caspase-3, and cleavage of PARP[56].

**2.12 *Ficus benghalensis* L.:** *Ficus benghalensis* L. is commonly known as 'Indian Banyan'. It is locally known as Bot (Bangladesh), Bargad (India), Gotu Nuga (Sri Lanka), and belongs to the family Moraceae. Although there was immuno-modulatory activity demonstrated using a methanol extract of *F. benghalensis* roots in an animal model[57], only an *in vitro* study exists in support of its anti-tumor activity of this plant. Hawary *et al.*[58] reported the methanol extract of *F. benghalensis* as a weak anti-tumor agent against HepG2 and MCF-7 tumor cell lines at a lower extant. However, this report might not represent the actual anti-cancer potential of *F. benghalensis* as suggested by the phyto-chemical constituent make up of *F. benghalensis*. Phyto-chemical analysis revealed the presence of several potential anti-cancer agents in different parts of plants especially in the leaves (i.e., lupeol, psoralen and $\beta$-sisterol)[59–62].

**2.13 *Ficus religiosa* L.:** *Ficus religiosa* L. is commonly known as 'Bo-tree', and belongs to the Moraceae family. It is locally known as Pan Bot (Bangladesh), Pipli (India), Bo (Sri Lanka) and Pippal (Pakistan). In a study, methanol and water extract of *F. religiosa* exhibited cyto-toxicity activity against HT29 and MDA-MB-435S cancer cells[12], but with more selective fractionation, the anti-cancer effect was increased in efficiency, as reported in a MCF-7 cell line[63]. *F. religiosa* contains a wide range of phyto-chemicals like phenols, amino acids, fatty acids, tannins, steroids, alkaloids and flavonoids are distributed throughout the different parts of plant. However, the anti-cancer activity specifically focuses on the several phyto-constituents including flavonols i.e. quercetin and myricetin, and phytosterols namely stigmasterol and $\beta$-sitosterol[64].

**2.14 *Hibiscus tiliaceus* Linn.:** *Hibiscus tiliaceus* Linn. is commonly known as 'Sea Hibiscus' and belongs to the Malvaceae family. It is locally known as Bhola (Bangladesh), Bilepatta (Sri Lanka), and Pola (India). *H. tiliaceus* roots have been reported to have anti-cancer activity against Dalton's Ascitic Lymphoma (DAL) in mice[65]. The cytotoxicy of an aqueous extract from *H. tiliaceous* leaves have also been demonstrated against gastric and colon cancer cells[12]. The various phyto-chemicals isolated from the plant are hibiscusin, hibiscus amide, vanillic acid, P-hydroxybenzoic acid, syringic acid, P-hydroxybenzaldehyde, scopoletin, N-tras-feruloyl-tyramine, N-cis-feruloyltyramine, $\beta$-sitosterol, stigmasterol, $\beta$-stigmasteronone, hibiscolactone, hibiscones, hibiscoqinones, lapachol, gossypol, gossypetin, manosonones, hyperoside, kaempferol, quercetin, gossypitin, gossytrine, para-coumaric and fumaric acid[66,67]. *H. tiliaceus* might possess a vast array of anti-cancer phyto-chemicals and might have potential to emerge due to the presence of known anti-cancerous photo-chemicals such as $\beta$-sitosterol, quercetin, kaempferol etc.

**2.15 *Jatropha gossypiifolia* L.:** *Jatropha gossypiifolia* is a bushy gregarious shrub belonging to the Euphorbiaceae Family and commonly known as 'Bellyache Bush'. It is locally known as Lal Bheranda (Bangladesh), Bhenenda (India), Rathu Erandu (Sri Lanka). Beyond the ethno-botanic use as an anti-cancer plant, there exist no direct evidence of anti-cancer activity of the crude extract of *J. gossypiifolia*, but phyto-chemicals of *J.*

**Figure 2.** Chemical structures of selected natural products **1**–**13**

*gossypiifolia* have been examined and are promising as anti-cancer agents. The reported phyto-chemical constituents include lignans and several diterpenoids, such as falodone (**9**) and jatrophone (**10**)[68,69]. Falodone has potent proliferation inhibitory activity in A-549 human cancer cell line[70], while Jatrophone exhibits anti-cancerous properties against P338 lymphocytic leukemia[71].

**2.16 *Kaempferia parviflora* Wall.:** *Kaempferia parviflora* Wall. Baker is a stemless, small, rhizomatous herb, commonly known as 'Thai Ginseng'. It is a member of the ginger family (Zingiberaceae), and is locally known as 'Kalahalood'. The ethanolic extract of *K. parviflora* rhizome is effective in suppressing the growth of HL-60 human leukemic cells[72]. This effect was found to be associated with the hallmarks of apoptotic cell death i.e., morphological cell change, loss in mitochondrial transmembrane potential and activation of caspase. Similar effect was observed using the crude ethanol extract of *K. parviflora* as reported in cholangio-carcinoma cell lines HuCCA-1 and RMCCA-1, where 5,7,4′-trimethoxyflavone (**11**) was identified as the anti-cancer bioactive component[73]. Moreover, 3,5,7,4′-tetramethoxyflavone, 5,7,4′-trimethoxyflavone and 5-hydroxy-3,7,3′,4′-tetramethoxyflavone purified from its rhizome extracts induces cytoxicity in human colorectal carcinoma (HCT-15) cells[74].

**2.17 *Lantana camara* L.:** *Lantana camara* L. is a species of flowering plant from the Verbenaceae family, and is commonly known as 'Spanish Flag'. The local name of *L. camara* is 'Urusia' in Bangladesh, 'Caturang' in India, and 'Gandapata' in Sri Lanka. Initally, the toxicity of *L. camera* in some animal species drew the attention of scientists, who revealed its anti-cancer effect[75]. Mahato *et al.*[76] isolated a novel flavonol glycoside, named camaraside (**12**), an anti-tumor agent from *L. camera*[76]. In addition, Sharma *et al.*[77] showed that lantadene A (**13**), a torpedoed component of *L. camera*, induced concentration and time-dependent inhibition of HL-60 cell proliferation by cell cycle arrest in the G0/G1 phase. Lantadene A also induced efficient HL-60 cell apoptosis by activating the caspase-3 pathway, and via the down- and up-regulation of Bcl-2 and Bax expression respectively[77].

**2.18 *Leea indica* (Burm.f.) Merr.:** *Leea indica* (Burm.f.) Merr., is a large shrub with ribbed branches and swollen nodes, belonging to the Leeaceae family. It is locally known as Kukur jiwa (Bangladesh), Kurkurjihva (India), Burulla (Sri Lanka) while its common name is 'Bandicoot Berry'. It was reported that methanol extract of *L. indica* slows the growth of EAC in Swiss albino mice, and reduces tumor weight[78]. The crude ethanol extract of *L. indica* and its ethyl acetate, hexane and water fractions showed cytotoxicity on various cancer cell lines, such as, Ca Ski, MCF 7, MDA-MB-435, KB, HEP G2 and WRL 68[79]. Two cycloartane triterpenoid glycosides, namely mollic acid arabinoside (MAA, **14**) and mollic acid xyloside (MAX, **15**) were isolated from *L. indica* with a concomitant demonstration of their anti-cancer effect. Both MAA and MAX are effective against the growth of cervical cancer cell line Ca Ski[80]. The cytotoxicity of MAA was characterized by both cytostatic and cytocidal effects. Specifically, MAA decreases the expression of proliferative cell nuclear antigen, increases sub-G1 cells, and arrest cells in S and G2/M phases[80].

**2.19 *Mollugo pentaphylla* Linn.:** *Mollugo pentaphylla* Linn., a perennial herb, is a member of Molluginaceae family that is commonly known as 'Itch Flower'. Its local names include 'Khetpapra (Bangladesh), and Jaraasi (India). In chemically induced model of carcinogenesis (DMBA is used as a carcinogen and TPA as a tumor initiator), methanol extract of *M. pentaphylla* showed significant anti-tumor activity[81]. As an ingredient of Peh-Hue-Juwa-Chi-Cao, traditional Chinese medicine preparations, *M. pentaphylla* exhibited anti-tumor activity against sarcoma-180 cells implanted subcutaneous tumors[82]. Several phyto-chemicals isolated from this plant include flavones such as apigenin and mollupentin, triterpenoids such as mollugogenol A, mollugogenol B, mollugogenol D, oleanolic acid and a steroid $\beta$-sitosterol[83], among which, apigenin, oleanolic acid, $\beta$-sitosterol have been implicated to possess an anti-cancer potential.

**2.20 *Murraya koenigii* Linn.:** *Murraya koenigii* Linn., is a plant belonging to the Rutaceae family and commonly known as 'Curry leaf tree'. The local name of *M. koenigii* are Bar Sunga (Bangladesh), Mitha neem (India), and Karapincha (Sri Lanka). Muthumani *et al.*[84] found that the non-polar extract of *M. koenigii* caused potent cytotoxic effect during short-term incubation (for 3 hours) in DAL cells. They have demonstrated that *M. koenigii* significantly decreases the viability and growth of DAL cells, when injected intraperitoneally into the mice, which was comparable to the effect of 5-flurouracil[84]. The alkaloids mahanine (**16**), mahanimbicine (**17**) and mahanimbine (**18**) were isolated from *M. koenigii,* and showed a significant anti-tumor activity against MCF-7, HeLa and P388 cell lines[85]. Mahanine induces the death receptor-mediated extrinsic pathway of apoptosis in MOLT-3 cancer cells, while also inducing the mitochondria dependent intrinsic pathway of apoptosis in HL-60[86,87]. Furthermore, 34 essential oils have been isolated from the leaves of *M. koenigii*, and all of which showed potent anti-bacteria and cytotoxic effects in a dose dependent trend[85]. These essential oil included 12 oxygenated monoterpenes, 12 sesquiterpene hydrocarbons, nine oxygenated sesquiterpenes and one oxygenated diterpene.

**2.21 *Nelumbo nucifera* Willd.:** The plant *Nelumbo nucifera* Willd., belongs to the family Nelumbonaceae, and popularly known as 'Sacred Lotus'. It is locally known as Kamal (India), Nelum (Sri Lanka), Nilufer (Pakistan) and and Padam (Bangladesh). During an *in vivo* study using the hydroethanolic extract of *N. nucifera*, there was noticable inhibition of growth and also the survival in mice who were intraperitonealy injected EAC cells, by modulating lipid peroxidation and augmenting antioxidant defense systems in EAC bearing mice[88]. Hemanth *et al.*[89] reported a similar effect of acetone extract of *N. nucifera* leaves in EAC bearing animals, and a significant cytotoxicity against HBL-100 *in vitro*. Also, the methanol extracts exhibited significant anti-proliferative effect in Calu-6 human pulmonary carcinoma and SMU-601 human gastric carcinoma cells[89]. Repeated reports confirm that the major constituents present in the *N. nucifera* are alkaloids (liensinine, neferine, pronuciferine, isoliensinine, negferine, asimilobine, nuciferine, remrefidine, isoliensinine etc.) and flavonoids, glycosylated flavonols, flavonol, aglycones (myricetin, quercetin, leucocyanidin, rutin, kaempferol, isorhamnetin, astragalin etc.)[90,91]. Neferine (**19**), a major alkaloid component in *N. nucifera* embryos, possessed a potent growth-inhibitory effect in human osteosarcoma cells, with a mechanism of p21-dependent cell cycle arrest at G1 due to p38 MAPK-mediated p21-stabilizing effect of neferine[91].

**2.22 *Nyctanthes arbor-tristis* L.:** *Nyctanthes arbour-tristis* L. is an evergreen shrub, commonly known as 'Binari' which is a member of Oleaceae family. Its local names are Sheuli (Bangladesh), Harsingar (India), Sepalika (Sri Lanka) and Srigading (Indonesia). Ethanolic extract of *N. arbor-tristis* stem bark showed significant anti-cancer activity against DAL in Swiss Albino mice[92]. Kumari *et al.*[93] observed a cytotoxic effect of a methanol extract of the dried leaf, fruit and stem in MDA-MB231 breast cancer cell line. To date, glycosides are found to be the most prominent phyto-chemical isolated from the petals, leaves, seeds and flowers of *N. arbor-tristis.* These glycosides include iridoid glycosides (arbortristosides A–C, nyctanthoside, arborside-C etc.), phenylpropanoid glycosides, carotenoid glucosides, phenylpropanoid glycoside (nyctoside A) and cardiac glycosides. Recent researches also reported the isolation of polysaccharides, $\beta$-sitosterol, $\beta$-amyrin, hentri-acontane, benzoic acid, nyctanthic acid, friedelin and lupeol and oleanolic acid and 6$\beta$-hydroxylonganin, alkaloids, phlobatanins, terpenoids from *N. arbor-tristis*[94,95].

**Figure 3.** Chemical structures of selected natural products **14**–**28**

**2.23 *Oxalis corniculata* Linn.:** *Oxalis corniculata* Linn., is a small creeping, low growing herbaceous plant, and is a member of the Oxalidaceae family, commonly known as 'Indian Sorrel'. It is locally known as Amrul (Bangladesh), Tipatia (India), Embul embiliya (Sri Lanka), Khatt-i-buti (Pakistan). The ethanolic extract of *O. corniculata* was found effective in inhibiting the tumor growth in EAC inoculated mice[96].

**2.24 *Physalis minima* L.:** *Physalis minima* L., a perennial herb which belongs to the Solanaceae family, and is commonly known as the 'Sun Berry''. It is locally known as Potka (Bangladesh) and Bandapariya (India). Studies repeatedly pronounced the striking anti-cancer effect of *P. minima* against several cancer cell lines. The chloroform extract of *P. minima* produced a significant growth inhibition in human T-47D breast carcinoma cell death via p53, caspase-3, and c-myc-dependent apoptotic pathways[97]. A combined effect of apoptotic and autophagic programmed cell death was found by chloroform extract of *P. minima* in cytotoxicity against Caov-3 cells[98]. The induction of this programmed cell death was mediated via c-myc, p53 and caspase-3 dependent pathway. The chloroform extract of *P. minima* also exhibited remarkable cytotoxic activities in NCI-H23 human lung adenocarcinoma cell line via apoptotic cell death[99]. Moreover, Ma *et al.*[100] isolated a panel of withanolides from this plant, which have moderate cytotoxic activity against human colorectal-carcinoma HCT-116 and human non small-cell lung-cancer NCI-H460 cell lines.

**2.25 *Pterocarpus santalinus* L.f.:** *Pterocarpus santalinus* L.f. belongs to the Fabaceae family, and commonly known as 'Red sandalwood'. Its local names are Rat handun (Sri Lanka), Lal-chandan (India), and Burada Sandal Safaid (Pakistan). Kwon *et al.* first documented the anti-cancer effect of *P. santalinus*[101]. Interestingly, in HeLa human cervical adenocarcinoma cell treated with a methanol extract of *P.*

*santalinus*, growth was inhibited and apoptosis induced, as confirmed a cell viability assay, chromatin condensation, DNA fragmentation and sub-G1 phase accumulation. Phyto-chemical investigations showed the presence of sesquiterpenes, isoflavones, lignans, and aurone glycosides[102]. Recently, Wu *et al.*[103] isolated five new benzofurans, pterolinuses A–E (**20**–**24**), six new neo-flavonoids, pterolinuses F–J, dehydromelanoxin, melanoxin (**25**), melanoxoin, S-3′-hydroxy-4,4′-dimethoxy-dalbergione and melannein from the extract of *P. santalinus* heartwood. Among the neoflavonoids, melanoxoin showed potent cytotoxicity against Ca9-22, *S*-3-hydroxy-4,4-dimethoxydalbergione showed cytotoxicity against Hep3B and MDA-MB-231, and pterolinus Hb exhibited cytotoxicity against A549 cancer cell lines[103]. Among the benzofurans, pterolinus-B showed cytotoxicity against A549 and MCF7 cell lines, while pterolinus-D showed cytotoxicity against Hep3B and MCF7 cell lines[103].

**2.26 *Salix tetrasperma* Roxb.:** *Salix tetrasperma* Roxb. (Family: Salicaceae), is commonly known as 'Indian Willow', Badah (India), and Burg Baid Sada (Pakistan). In a study, Islam *et al.*[104] observed the *in vivo* cytotoxic activity of the organic extract of *S. tetrasperma* against EAC tumor bearing mice. Phyto-chemical studies of *S. tetrasperma* resulted in the isolation of several tannins, triterpenes, viz. *β*-amyrin, lupeol, chalcinasterol, steroids (viz. *β*-sitosterol and stigmasterol)[105].

**2.27 *Solanum nigrum* Linn.:** *Solanum nigrum* Linn. belongs to the Solanaceae family, and commonly known as Black Nightshade. It is locally known as Kack-machi (Bangladesh), Bandakh (India), Kalu Kamberiya (Sri Lanka), and Ab Makoh (Pakistan). Extracts from *S. nigrum* was found to induce a strong cytotoxic effect in HepG2 hepatoma cells and AU565 breast cancer cells by inducing apoptosis and/or autophagocytosis[106,107]. Actually, a low dose treatment induced only autophagy, while high dose treatment induced both of autophagy and apoptosis. Although various parts of the *S. nigrum* plant contain polyphenols, gentisic acid, luteolin, apigenin, kaempferol, *m*-coumaric acid, anthocyanidin, their role in apoptosis is not clear. The crude polysaccharides from *S. nigrum* concomitantly up-regulates pro-appototic gene Bax and down-regulates Bcl-2 and mutant p53 gene expression in mice bearing cervical cancer (U14)[108]. On the other hand, pepsin and pancreatin digestion resistant peptide Lunasin has been claimed as a bioactive and bioavailable component due to inhibition of H3 and H4 acetylation and the phosphorylation of the Rb protein in mammalian cells and in a skin cancer mouse model[109].

**2.28 *Tinospora crispa*:** *Tinospora crispa* is an indigenous climber plant that is locally known as Akar Patawali in Bangladesh and Geta Kinda in Sri Lanka. It is a member of the Menispermaceae family. Several studies suggested the efficient cytoxicity of *T. crispa* polar extracts. Aqueous crude extract of *T. crispa* was treated against MCF-7, HeLa, HepG2 and Caov-3 cancer cell lines, using cisplatin and tamoxifen as standard in a study by Zulkhairi *et al.*[110]. The extract exhibited anti-proliferative activity in all cancer cell lines, which was comparable with standard drugs[110]. Ibahim *et al.*[111] also treated several cancer cell lines (MCF-7, MDA-MB-231, HeLa and 3T3) with water, methanol or chloroform extract of *T. crispa* stem, and reported a dose-dependent anti-proliferative activity against all cancerous cell lines with the most potent effect of methanol extract on MDA-MB-231 and MCF-7[111]. The antiproliferative effect of *T. crispa* might be due to its chemical substances especially alkaloids, which are known to have anti-cancer properties. Several alkaloids have been isolated, namely tinoscorside A (**26**), N-formylanonaine (**27**), N-formyldehydroanonaine (**28**), N-formylnomuciferine (**29**), N-demethyl-N-formyldehydronornuciferine (**30**), magnoflorine (**31**), paprazine (**32**), N-trans-feruloyltyramine (**33**), N-formylasimilobine 2-*O*-*β*-D-glucopyranoside (**34**) from *T. crispa*[112].

**2.29 *Tragia involucrate* L.:** *Tragia involucrata* L. is a member of the Euphorbiaceae family, and commonly known as 'Stinging Nettle'. It is locally known as Bichuti (Bangladesh), Barhanta (India), Wel Kahambiliya (Sri Lanka). *T. involucrate* was found to have anti-cancer activity both *in vitro* and *in vivo* model system. The hexane and ethyl acetate extracts of *T. involucrata* significantly increased the life span of EAC baring mice in a dose dependant manner[113]. Phyto-chemical analysis of *T. involucrata* showed the presence of flavonoids and terpenoids that were claimed to have possess anti-cancer activity, and probably explain the cytotoxic effect of *T. involucrate* extract[114].

**2.30 *Vitex negundo* L.:** *Vitex negundo* L., commonly known as 'Chaste tree', belongs to the Verbenaceae family. It is locally known as Nishinda (Bangladesh), Nika (Sri Lanka), Bana (India), Sambhalu (Pakistan). Ethanolic extract of *V. negundo* leaf showed significant anti-tumor activity against DAL bearing Swiss albino mice[115]. This extract was also found to be cytotoxic against mouse lung fibroblast (L-929) cells in long term chemosensitive cytotoxic assay[115]. Saluja *et al.*[116] also demonstrated cytotoxic activity of acetone and ethanol extracts of *V. negundo* leaves in EAC bearing mice. A known flavone, vitexicarpin was isolated from the chloroform-soluble extract of the *V. negundo* leaves that exhibited broad cytotoxicity in a human cancer cell line panel[117]. Zhou *et al.*[118] isolated a series of lignan compounds known as Vitexins from the seed of *V. Negundo*, and named them as EVn-50. EVn-50 was found to contain mainly VB1 (6-hydroxy-4-(4-hydroxy-3-methoxyphenyl)-3-hydroxymethyl-7-methoxy-3,4-dihydro-2-naphthaldehyde, **35**) and VB2 (6-hydroxy-4-(4-hydroxy-3-methoxyphenyl)-3-hydroxymethyl-5-methoxy-3,4-dihydro-2-naphthaldehyde, **36**). They showed cytotoxic effect on breast, prostate and ovarian cancer cells by inducing apoptosis through pro-apoptotic process, which is mediated by a decreased Bcl-2/Bax ratio and activation of caspases[118].

## 3 Concluding Remarks

The actual contribution of South Asia toward the anti-cancer research seems to be deemed and negligible. Being third world countries, research facilities are limited, whereas natural resourses are abundant. The variation in geography and environment of this region provides a rich plant biodiversity, many of which can be used as the source of anti-cancer agents. As the use of phyto-chemicals in cancer treatment is promising and increasing rapidly, the proper scientific study to extract chemicals, investigation of their anti-cancer role in details, and clinical studies might be the interesting subject of future cancer research.

**Figure 4.** Chemical structures of selected natural products **29–36**

**Open Access** This article is distributed under the terms of the Creative Commons Attribution License which permits any use, distribution, and reproduction in any medium, provided the original author(s) and source are credited.
